# GLUD1 supports ovarian cancer progression by counteracting anoikis via ARAF/MEK/ERK signaling

**DOI:** 10.1038/s41698-026-01349-6

**Published:** 2026-03-05

**Authors:** Huolun Feng, Yanzhen Chen, Geyan Wu, Zhentao Zhang, Hongkun Lai, Changnian Yang, Shaofen Zhang, Yongqing Lin, Yingqi Liu, Haiyan Ye, Shanshan Wu, Lixue Cao

**Affiliations:** 1 Guangdong Cardiovascular Institute, Guangdong Provincial People’s Hospital (Guangdong Academy of Medical Sciences), Guangzhou, Guangdong China; 2 Department of Gastrointestinal Surgery, Department of General Surgery, Guangdong Provincial People’s Hospital (Guangdong Academy of Medical Sciences), Southern Medical University, Guangzhou, China; 3 Department of Gynecology, Guangdong Provincial People’s Hospital, Guangdong Academy of Medical Sciences, Southern Medical University, Guangzhou, Guangdong China; 4 Biomedicine Research Centre, The Third Affiliated Hospital of Guangzhou Medical University, Guangzhou Medical University, Guangzhou, China; 5 The First School of Clinical Medicine, Guangdong Medical University, Zhanjiang, Guangdong China; 6 Department of Biology, School of Basic Medical Sciences, Guangdong Medical University, Zhanjiang, Guangdong China; 7 Medical Research Institute, Guangdong Provincial People’s Hospital (Guangdong Academy of Medical Sciences), Southern Medical University, Guangzhou, Guangdong China

**Keywords:** Cancer, Oncology

## Abstract

Peritoneal dissemination is the major cause of mortality in epithelial ovarian cancer (EOC) and requires tumor cells to survive in a detached state by evading anoikis. However, the molecular mechanisms supporting anchorage-independent survival remain poorly defined. Here, we identify glutamate dehydrogenase 1 (GLUD1) as a key regulator of anoikis resistance and metastatic progression in EOC. GLUD1 expression was elevated in metastatic EOC tissues and associated with unfavorable clinical outcomes. Loss of GLUD1 impaired anoikis resistance and reduced metastatic capacity of ovarian cancer cells in vitro, while markedly suppressing peritoneal dissemination and prolonging survival in vivo. Mechanistically, GLUD1 was found to interact with a key protein ARAF, the A-Raf proto-oncogene. By limiting ubiquitin–proteasome-mediated degradation of ARAF, GLUD1 exerted a non-enzymatic function that stabilized ARAF protein levels and sustained MEK/ERK signaling.

Together, these findings reveal a non-canonical role of GLUD1 in regulating protein stability and identify the GLUD1–ARAF axis as a critical mechanism supporting anchorage-independent survival during peritoneal dissemination of EOC.

## Introduction

According to GLOBOCAN 2020, ovarian cancer remains the most lethal gynecologic malignancy, with epithelial ovarian cancer (EOC) accounting for the majority of cases and deaths^1^. Its high mortality is largely attributed to extensive peritoneal metastasis and the presence of malignant ascites at diagnosis^2^. Within malignant ascites, EOC cells aggregate in suspension and survive in an anchorage-independent manner by evading anoikis, a form of programmed cell death triggered by detachment from the extracellular matrix (ECM)^3,4^. Although anoikis resistance is essential for metastatic dissemination, the molecular mechanisms that sustain the survival of detached EOC cells remain incompletely understood.

Metabolic reprogramming has emerged as a key adaptive strategy that enables cancer cells to evade anoikis and support metastatic progression^5,6^. Alterations in glycolysis, amino acid metabolism, and lipid metabolism collectively meet the energetic and biosynthetic demands of detached tumor cells^6–8^. Increasing evidence suggests that glutamine metabolism contributes to the maintenance of tricarboxylic acid (TCA) cycle activity under anchorage-independent conditions, underscoring the metabolic flexibility required for survival during detachment^6,9,10^. Glutamate dehydrogenase 1 (GLUD1), a key enzyme in glutamine metabolism, catalyzes the conversion of glutamate to α-ketoglutarate (α-KG), supporting TCA cycle replenishment to meet the energy and biosynthetic needs of detached tumor cells^11–15^. However, the specific role of GLUD1 in anoikis resistance and metastatic progression of EOC remains poorly defined.

In this study, we integrated transcriptomic datasets with curated gene signatures related to anoikis and amino acid metabolism and identified GLUD1 as a candidate gene associated with metastatic progression. GLUD1 expression was markedly elevated in metastatic EOC tissues and correlated with shorter overall and disease-free survival. Functional studies demonstrated that GLUD1 critically promotes anoikis resistance and metastasis in vitro and in vivo. Notably, our findings reveal that GLUD1 exerts a previously unrecognized non-enzymatic regulatory function by modulating the stability of the RAF family kinase ARAF, thereby sustaining MEK–ERK signaling under detachment conditions. These results reveal an unappreciated mechanism through which GLUD1 contributes to anchorage independent survival and provide new insight into the metastatic biology of EOC.

## Results

### GLUD1 is identified as an anoikis- and amino acid metabolism–related gene in EOC

**Fig. 1:**
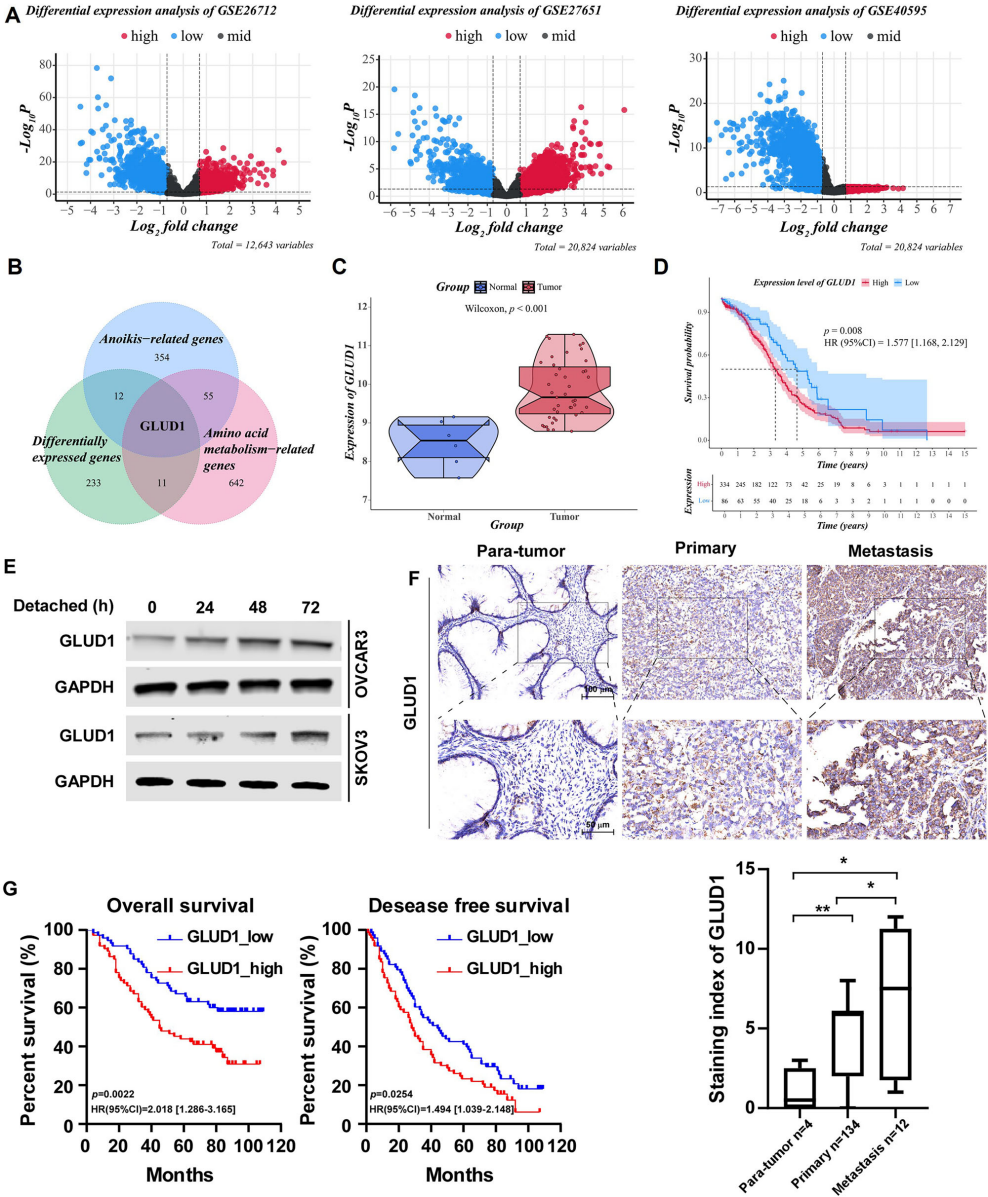
Involvement of GLUD1 in anoikis resistance and metastasis of EOC. **A** Volcano plot of GLUD1 expression in epithelial ovarian cancer (EOC) from GEO datasets. Colors indicate gene expression levels: red for high, blue for low, and gray for non-significant expression changes. **B** Venn diagram illustrating the overlap among DEGs, anoikis-related genes, and amino acid metabolism–related genes. **C** GLUD1 expression in the GSE27651 dataset. Gene expression levels are shown with red representing tumor tissues and blue representing normal controls. **D** Kaplan-Meier curves showing overall survival differences between high and low GLUD1 expression groups in EOC patients from the TCGA dataset. Red and blue lines represent high and low expression levels, respectively. **E** Western blot analysis of GLUD1 expression in OVCAR3 and SKOV3 cells cultured under detached conditions. GAPDH was used as a loading control. **F** Representative immunohistochemistry images (Upper) and quantification (Lower) of GLUD1 expression in para-tumor, primary, and metastatic EOC tissues. **G** Overall survival curve and disease-free survival curve for EOC patients with low or high expression of GLUD1 based on IHC staining index. Data are presented as mean ± SD. *p < 0.05, **p < 0.01.

To identify key regulators in ovarian cancer, we performed a comprehensive analysis of three publicly available gene expression datasets (GSE26712, GSE27651, and GSE40595; total n = 278). Differentially expressed genes (DEGs) between EOC tissues and controls were identified using predefined statistical criteria with criteria of false discovery rate (FDR) < 0.05 and |log₂FC | ≥ 0.7 (Fig. 1A). These genes were subsequently intersected with curated gene sets related to anoikis and amino acid metabolism. Among all candidates, GLUD1 was the only gene present in both functional gene sets (Fig. 1B). Other genes, such as GLS2, SLC1A5, and SLC7A5, were found in only one of the two functional gene sets and were therefore not pursued further. Analysis revealed that GLUD1 expression was significantly upregulated in ovarian cancer tissues compared to normal controls (GSE27651, *p* < 0.001, Fig. 1C). Furthermore, Kaplan-Meier survival analysis demonstrated that elevated GLUD1 expression was significantly associated with worse overall survival (OS) and progression-free survival (PFS) in EOC patients (TCGA-OV, n = 420, *P* = 0.008; KM-plotter, n = 373, *p* = 0.0045; KM-plotter, n = 177, *p* = 0.044; Fig. 1D and Supplementary Fig. 1A).

Further stratified analysis revealed that the inverse association between GLUD1 expression and progression-free survival was significant in patients with advanced-stage disease (stage III/IV), whereas no significant association was observed in early-stage patients (stage I/II) (KM-plotter OV-I/II, n = 163, p = 0.27; OV-III/IV, n = 1081, p = 0.049; Supplementary Fig. 1B). Collectively, these analyses identify GLUD1 as a gene associated with anoikis, amino acid metabolism, and unfavorable prognosis in advanced-stage epithelial ovarian cancer.

### GLUD1 expression is elevated in metastatic EOC and correlates with poor clinical outcomes

Based on a screening of GLUD1 protein expression, we chose the OVCAR3 and SKOV3 ovarian cancer cell lines for subsequent experiments. (Supplementary Fig. 2). To assess whether GLUD1 expression is regulated during anoikis, OVCAR3 and SKOV3 cells were cultured under detached conditions. GLUD1 protein levels increased progressively during suspension culture, consistent with a potential role in anoikis resistance (Fig. 1E).

In view of the results, immunohistochemical analysis was performed on the clinical specimens of ovarian cancer. It was found that GLUD1 expression was significantly higher in metastatic epithelial ovarian cancer tissues than in primary tumors and adjacent non-tumor tissues (Fig. 1F). Based on immunohistochemical scoring, patients were stratified into GLUD1_high and GLUD1_low groups. Kaplan-Meier analysis revealed that high GLUD1 expression was associated with shorter overall survival (*p* = 0.002) and disease-free survival (*p* = 0.02) (Fig. 1G). Clinical correlation analysis showed that GLUD1 expression was significantly associated with patients’ vital status, with a higher proportion of deaths in the high-expression group (Supplementary Table 1). Both univariate and multivariate Cox regression analyses identified GLUD1 as an independent risk factor for overall survival (Supplementary Table 2). Together, these findings indicate that GLUD1 expression is elevated in metastatic epithelial ovarian cancer and correlates with adverse clinical outcomes.

### GLUD1 dysregulation drives anoikis resistance and migration in EOC cells

**Fig. 2:**
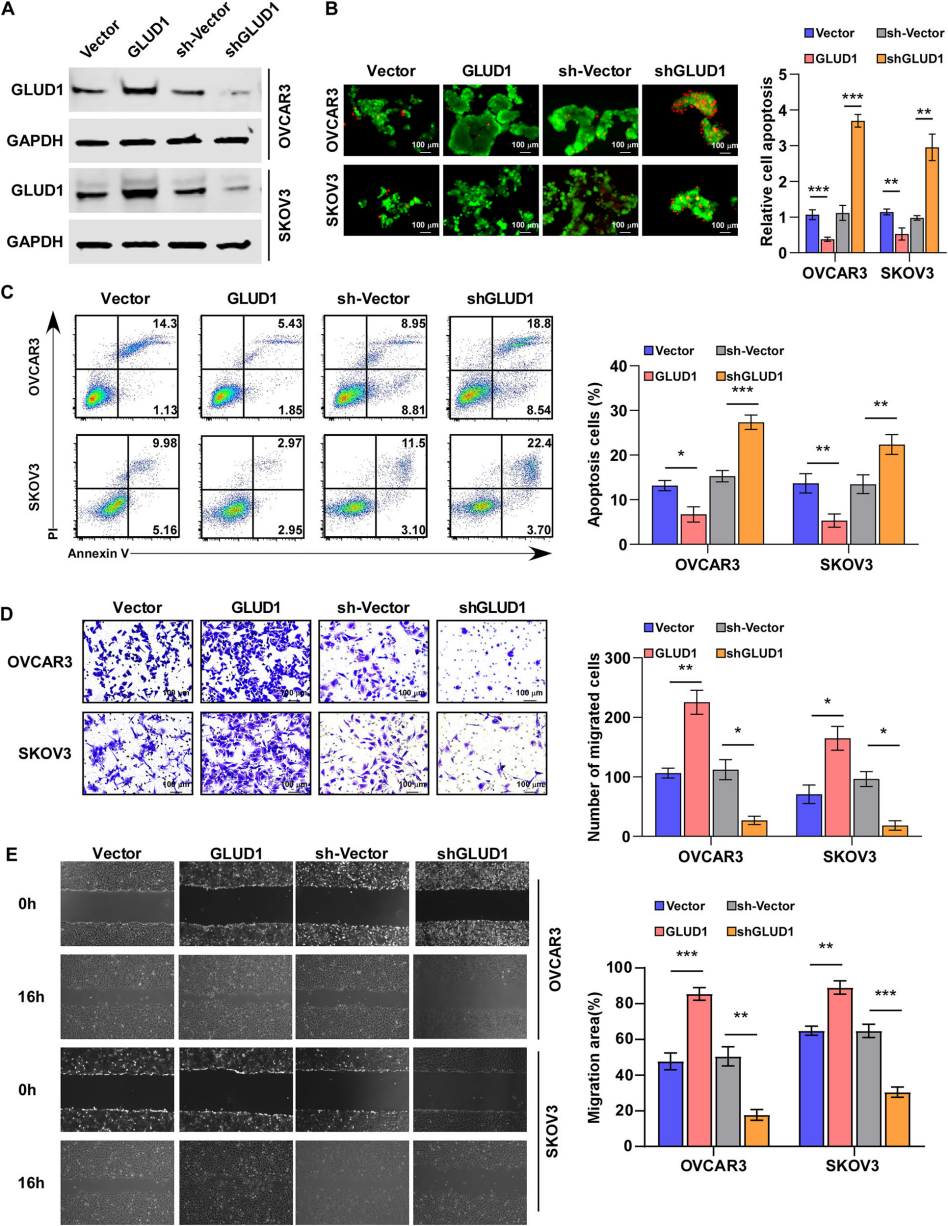
GLUD1 dysregulation drives anoikis resistance and migration in EOC cells. **A** Western blot analysis of GLUD1 expression in ovarian cancer cells with GLUD1 overexpression or knockdown. **B** Representative fluorescence images of live (green) and dead (red) cells under detached conditions (left) and quantification of live dead cell ratios (right). **C** Flow cytometric analysis of apoptosis in ovarian cancer cells cultured under detached conditions and corresponding quantification. **D** Transwell migration assays of SKOV3 and OVCAR3 cells with GLUD1 overexpression or knockdown and quantification of migrated cells. **E** Wound healing assays assessing migratory capacity of SKOV3 and OVCAR3 cells and quantification of wound closure. All experiments were performed in three independent biological replicates. Data are presented as mean ± SD. *p < 0.05, **p < 0.01, ***p < 0.001.

To investigate the functional role of GLUD1 in epithelial ovarian cancer, stable GLUD1-overexpressing and GLUD1-silenced OVCAR3 and SKOV3 cell lines were established (Fig. 2A). Under suspension culture conditions, GLUD1 knockdown resulted in a time-dependent reduction in spheroid size and proliferative capacity (Supplementary Fig. 3). Calcein-AM/PI staining demonstrated that GLUD1 overexpression reduced apoptosis, whereas GLUD1 silencing caused a significant, time-dependent increase in apoptosis under detached conditions (Fig. 2B). These results were corroborated by Annexin V–FITC/PI flow cytometry analysis, which similarly showed that GLUD1 overexpression suppressed, while GLUD1 knockdown enhanced, anoikis in suspension cultures (Fig. 2C).

Given the established link between anoikis resistance and metastatic dissemination, we next examined whether GLUD1 influences cell migration. Gene set enrichment analysis revealed that GLUD1 expression positively correlated with gene signatures associated with migration and metastasis (Supplementary Fig. 4A). Consistently, GLUD1 overexpression enhanced, whereas GLUD1 knockdown impaired, the migratory capacity of epithelial ovarian cancer cells in both Transwell and wound-healing assays (Fig. 2D, E). These findings demonstrate that GLUD1 promotes anoikis resistance and migratory behavior in epithelial ovarian cancer cells.

### Knockdown of GLUD1 suppressed peritoneal dissemination in vivo

**Fig. 3:**
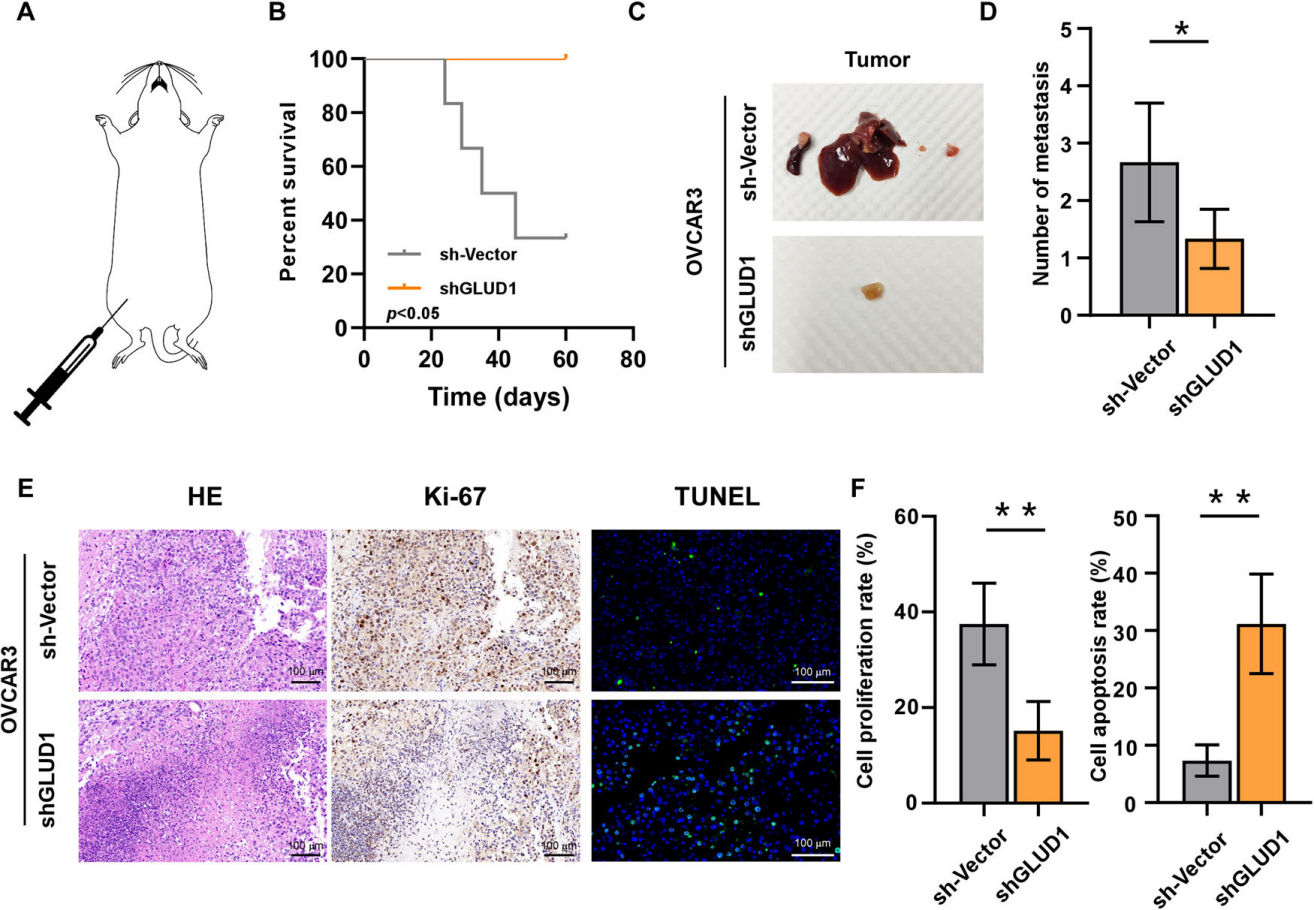
GLUD1 inhibition suppresses peritoneal dissemination in vivo. **A** Establishment of an intraperitoneal ovarian cancer implantation mouse model. **B** Survival curves of tumor bearing mice injected with control or GLUD1 silenced cells. **C** Representative images of peritoneal metastatic nodules. **D** Quantification of metastatic nodules per mouse. **E** Representative images of H&E, Ki-67, and TUNEL staining of tumor tissue sections. **F** Quantification of Ki 67 positive cells and TUNEL positive cells. n = 6 mice per group. Data are presented as mean ± SD. *p < 0.05, **p < 0.01.

To further investigate the in vivo biological role of GLUD1, a mouse model of intraperitoneal ovarian cancer implantation was established (Fig. 3A). Survival analysis revealed that GLUD1 inhibition significantly prolonged the survival of tumor-bearing mice compared to the control group (Fig. 3B). Upon necropsy, a notable reduction in metastatic nodules in the spleen, liver, and peritoneal cavity was observed in the GLUD1-silenced group (Fig. 3C, D). Histological analysis of abdominal tumor tissues, including Hematoxylin and Eosin (H&E) and TUNEL staining, demonstrated that tumors derived from GLUD1-silenced cells exhibited a higher proportion of apoptotic cells (Fig. 3E, F). Immunohistochemistry (IHC) further confirmed reduced GLUD1 expression, and importantly, a decrease in Ki-67 staining, indicating reduced proliferative activity in the GLUD1 knockdown group (Fig. 3E, F). Collectively, these in vivo results demonstrate that GLUD1 suppression inhibits tumor growth, survival, and peritoneal dissemination in ovarian cancer.

### GLUD1 interacts with and stabilizes ARAF to activate MEK–ERK signaling

**Fig. 4:**
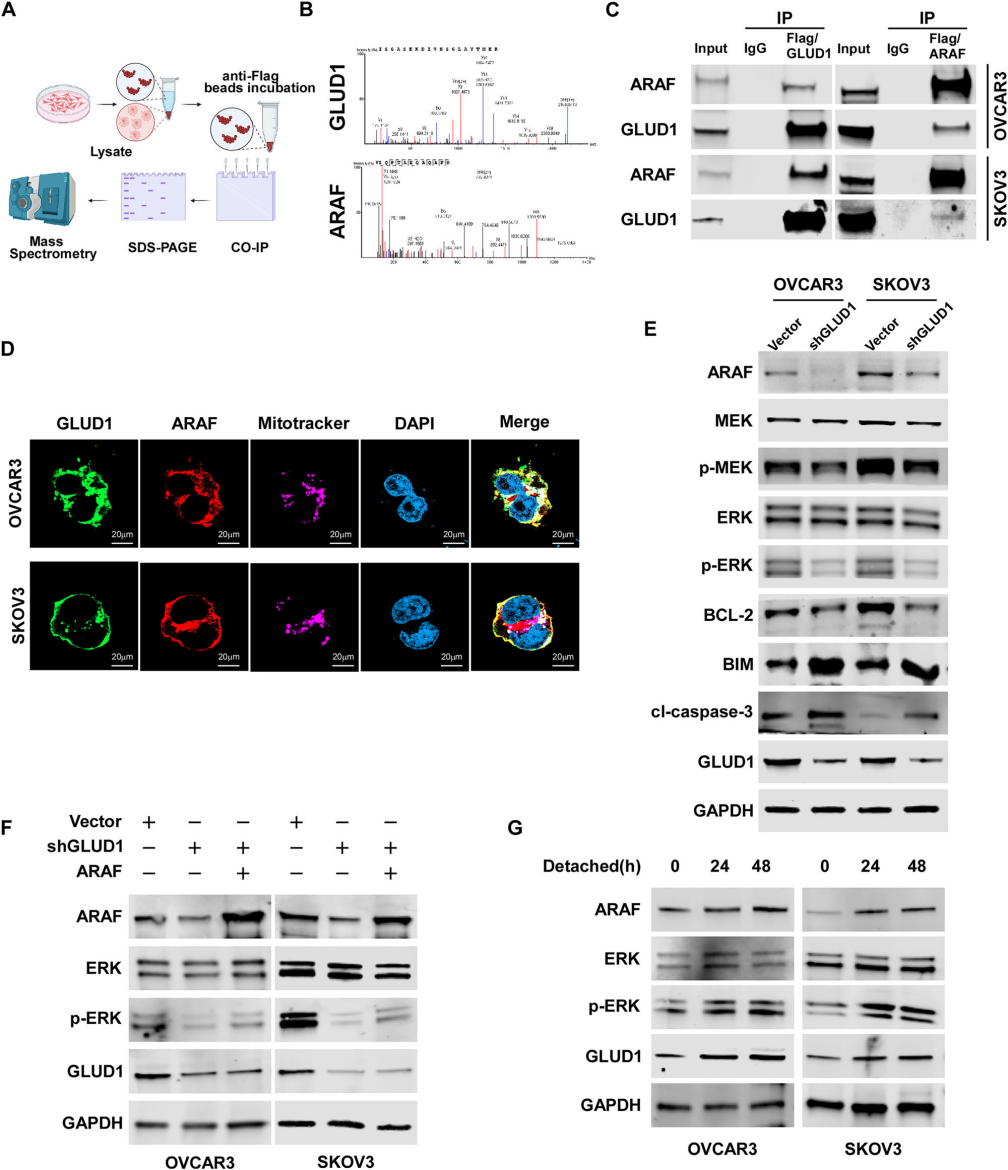
GLUD1 interacts with ARAF and activates MEK/ERK signaling. **A** Mass spectrometry analysis identifying candidate GLUD1 interacting proteins in GLUD1 overexpressing cells. Created in BioRender. cao, S. (2026) https://BioRender.com/yvkjnco. **B** Peptide spectra supporting the interaction between GLUD1 and ARAF. **C** Co immunoprecipitation analysis of GLUD1 and ARAF in cells expressing the indicated cells. **D** Immunofluorescence images showing the subcellular localization of GLUD1 and ARAF with mitochondrial staining. Scale bars, 20 μm. **E** Western blot analysis of ARAF, MEK, phosphorylated-MEK, ERK, phosphorylated-ERK, BCL-2, BIM, cleaved-caspase 3 in the indicated cells. GAPDH was used as a loading control. **F** Western blot analysis showing rescue of ARAF expression and ERK activation by ARAF re-expression in GLUD1 silenced cells. **G** Time course analysis of ARAF and MEK/ERK signaling under detached conditions in ovarian cancer cells. GAPDH was used as a loading control.

GSEA analysis revealed that GLUD1 expression is positively associated with RAF activation and ERK/MAPK signaling pathways (Supplementary Fig. 4B). To elucidate the molecular mechanisms underlying GLUD1 function in ovarian cancer cells, IP-MS was performed in OVCAR3-GLUD1-overexpressing cells, leading to the identification of GLUD1-interacting proteins (Fig. 4A). Among the specific interactors, ARAF was identified as a binding partner of GLUD1, and is known to modulate survival signaling (Fig. 4B). We further performed immunoprecipitation (IP) assays to evaluate the interaction between GLUD1 and ARAF. The result showed that GLUD1 is bound to ARAF (Fig. 4C). Subcellular localization analyses showed that ARAF predominantly co-localizes with GLUD1 in the cytoplasm, with only a minor fraction in mitochondria (Fig. 4D). Interestingly, knockdown of GLUD1 led to a pronounced reduction in ARAF protein levels, whereas BRAF and CRAF expression remained unaffected and neither interacted with GLUD1 (Fig. 4E and Supplementary Fig. 5A, B). Given that ARAF is reported to activate the MEK/ERK pathway and modulate apoptosis in cancer cells, we next examined this signaling axis. Western blot confirmed that GLUD1 knockdown significantly inhibited ARAF levels and phosphorylation of MEK and ERK. Concomitantly, expression of the anti-apoptotic protein BCL-2 decreased, whereas levels of BIM and cleaved caspase-3 increased (Fig. 4E). Moreover, re-expression of ARAF restored ERK phosphorylation in cells with GLUD1 knockdown (Fig. 4F). Consistently, under suspension conditions, cells with higher GLUD1 levels exhibited a time-dependent increase in ARAF and p-ERK, indicating an adaptive survival response activated to counteract ongoing anoikis stress (Fig. 4G). Collectively, these results suggest that GLUD1 specifically stabilizes ARAF and promotes downstream MEK/ERK signaling to regulate cell survival.

### GLUD1 stabilizes ARAF by suppressing ubiquitin–proteasome–mediated degradation

**Fig. 5:**
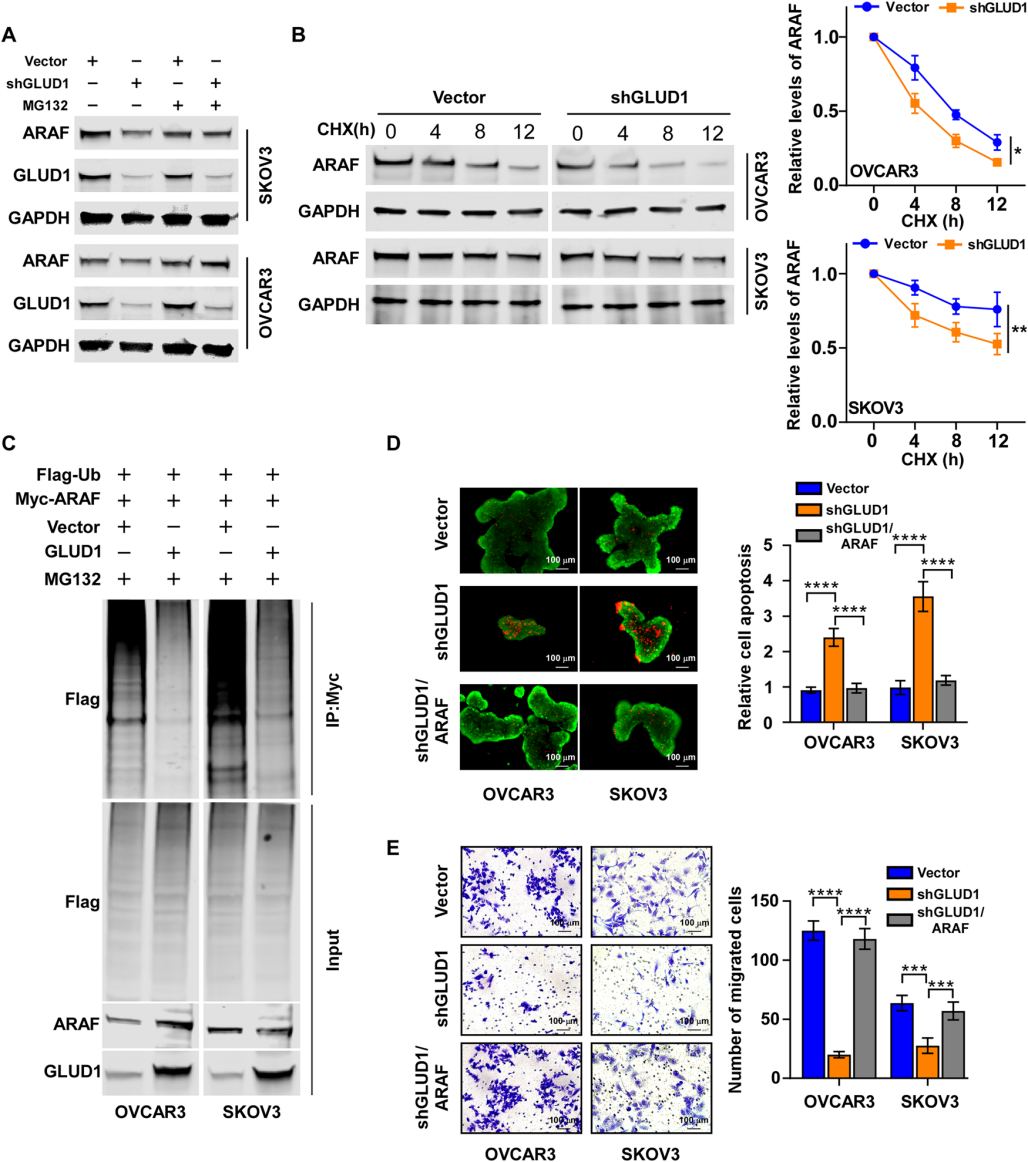
GLUD1 Stabilizes ARAF by Suppressing Ubiquitin Proteasome Mediated Degradation. **A** Western blot analysis of ARAF protein levels in cells treated with the proteasome inhibitor MG132. **B** Western blot analysis of ARAF protein levels in GLUD1-silenced cells treated with cycloheximide (CHX; 25 μg/mL) for 0, 4, 8, and 12 h (left). The decay curve of ARAF, normalized to GAPDH and to the 0-hour time point, is shown on the right. **C** Immunoprecipitation of ARAF followed by immunoblotting for ubiquitin to assess ARAF ubiquitination in the indicated cells. **D** Live dead cell staining of detached ovarian cancer cells following GLUD1 knockdown with or without ARAF re-expression and quantification. **E** Transwell migration assays assessing the effects of ARAF re expression on migration of GLUD1 silenced cells and quantification. Data are presented as mean ± SD from three independent experiments. *p < 0.05, **p < 0.01, ***p < 0.001, ****p < 0.0001.

To further investigate the mechanism by which GLUD1 regulates ARAF expression, we examined whether ARAF protein stability is controlled by proteasomal degradation. Treatment with the proteasome inhibitor MG132 reversed the reduction of ARAF protein levels induced by GLUD1 knockdown, indicating that GLUD1 regulates ARAF at the post-translational level (Fig. 5A). Consistently, cycloheximide chase assays revealed that the half-life of ARAF was significantly shortened in GLUD1-silenced cells compared with control cells (Fig. 5B). To determine whether ubiquitin–proteasome–mediated degradation underlies GLUD1-dependent ARAF stabilization, ubiquitination assays were performed. Ubiquitination assays revealed decreased polyubiquitination of ARAF upon GLUD1 overexpression, supporting a role for the ubiquitin–proteasome pathway in this process (Fig. 5C). To functionally validate this regulatory relationship, ARAF was reintroduced into GLUD1-silenced ovarian cancer cells. Re-expression of ARAF partially rescued the increased apoptosis observed under detached conditions following GLUD1 knockdown (Fig. 5D). In addition, Transwell migration assays demonstrated that ARAF re-expression restored the impaired migratory capacity induced by GLUD1 depletion (Fig. 5E). Collectively, these results indicate that GLUD1 promotes anoikis resistance and metastatic behavior in ovarian cancer cells by stabilizing ARAF protein.

### Clinical correlation of GLUD1 and ARAF expression in EOC

**Fig. 6:**
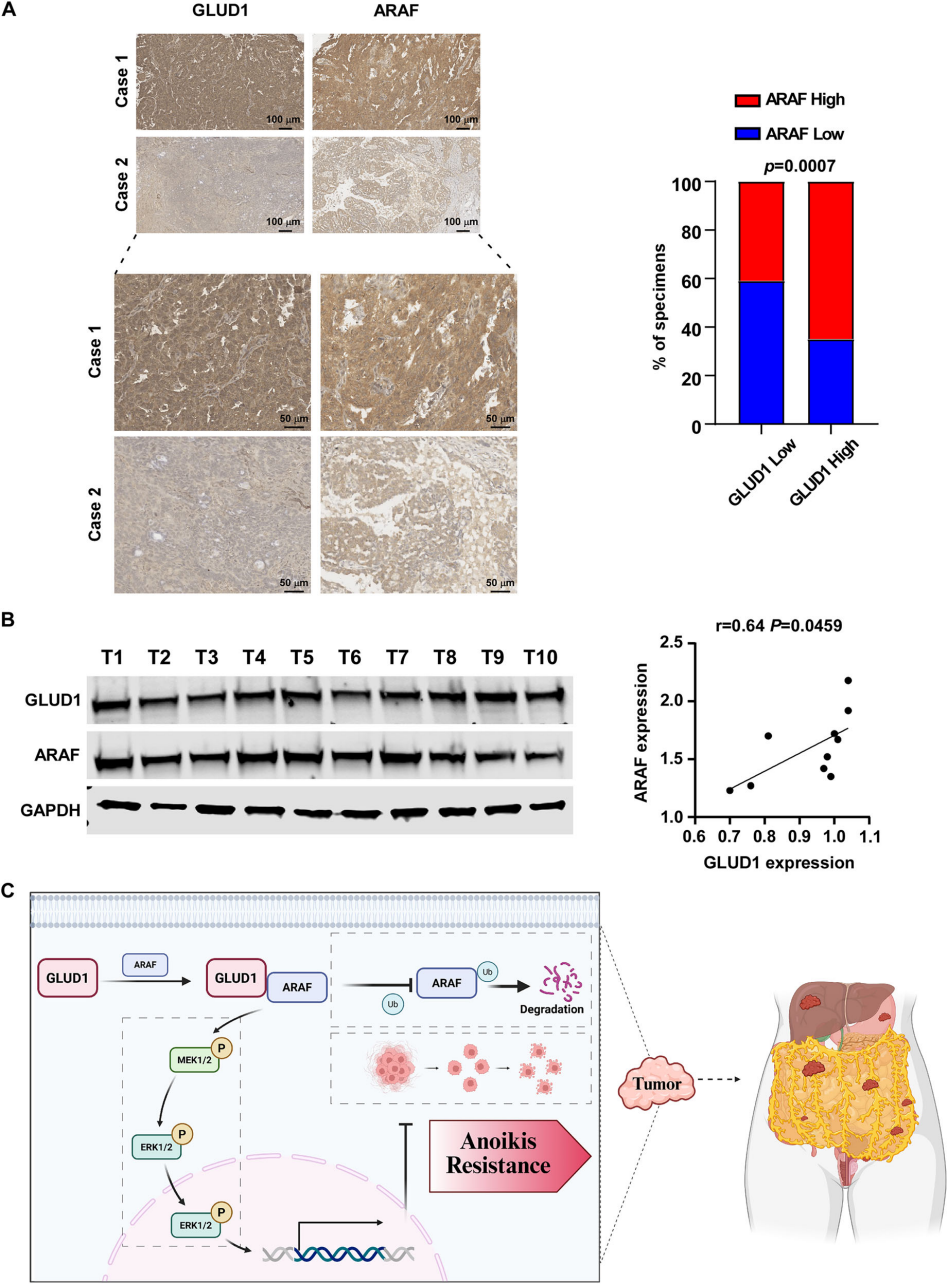
GLUD1 expression correlates with ARAF in clinical EOC specimens. **A** Representative immunohistochemical (IHC) staining images of GLUD1 and ARAF in EOC tissues (left). Correlation analysis of GLUD1 and ARAF protein expression levels in clinical EOC samples (right). **B** Western blot analysis of GLUD1 and ARAF in 10 freshly collected EOC tissue samples (left). Linear regression analysis demonstrating their correlation (right). **C** Schematic model illustrating GLUD1 mediated stabilization of ARAF and activation of MEK/ERK signaling to promote anoikis resistance and metastatic dissemination in EOC. Created in BioRender. cao, S. (2026) https://BioRender.com/8awa5ej.

To validate the relevance of the GLUD1–ARAF axis in clinical specimens, GLUD1 and ARAF protein expression was examined in epithelial ovarian cancer tissues. Immunohistochemical analysis revealed a significant positive correlation between GLUD1 and ARAF expression levels (Fig. 6A). This association was further confirmed in freshly collected ovarian cancer specimens, which exhibited concordant expression patterns of GLUD1 and ARAF (Fig. 6B). Collectively, these findings support a model in which GLUD1 promotes anoikis resistance and metastatic progression in epithelial ovarian cancer through stabilization of ARAF and activation of downstream MEK–ERK signaling (Fig. 6C).

## Discussion

Detached EOC cells encounter significant metabolic and signaling stress within malignant ascites, and their ability to evade anoikis is therefore essential for successful peritoneal dissemination^1–3^. This biological context underscores the need to define the molecular processes that permit anchorage-independent survival. In this study, we identify GLUD1 as a determinant of anoikis resistance in EOC. GLUD1 expression is elevated in metastatic tissues, correlates with unfavorable clinical outcomes, and is required for the survival and dissemination of detached tumor cells. Together, these findings address an important knowledge gap regarding the mechanisms that sustain epithelial ovarian cancer cell viability during transcoelomic spread and reveal a link between amino acid metabolism–associated genes and survival signaling in this setting.

Accumulating evidence indicates that GLUD1 can influence oncogenic signaling pathways, suggesting that its biological functions may extend beyond canonical metabolic regulation^11–15^. For example, GLUD1 promotes metastasis of EGFR-activated lung cancer through activation of cAMP response element-binding protein (CREB) signaling^15^, and α-ketoglutarate generated by GLUD1 suppresses H3K27 methylation, leading to upregulation of PDPK1 and activation of the PI3K/AKT/mTOR pathway^14^. Despite these observations, the mechanisms by which GLUD1 regulates survival signaling in epithelial ovarian cancer have remained unclear. In this study, using immunoprecipitation–mass spectrometry followed by co-immunoprecipitation validation, we identify ARAF as a novel binding partner of GLUD1. This interaction enhances ARAF protein stability, thereby activating MEK/ERK signaling and promoting anoikis resistance and metastatic behavior in ovarian cancer cells.

GLUD1 has been classically characterized as a metabolic enzyme that catalyzes the conversion of glutamate to α-ketoglutarate, thereby regulating glutaminolysis and influencing tumor cell proliferation, apoptosis, and metastasis^11,12,14,15^. Consistent with this enzymatic role, our data show that α-ketoglutarate supplementation partially restores ARAF abundance and ERK activity (Supplementary Figs. 6A–B). However, the incomplete rescue indicates that GLUD1-mediated regulation of ARAF cannot be fully explained by its catalytic activity alone. These findings support a non-enzymatic role for GLUD1 in sustaining survival signaling under detachment conditions. In line with this concept, GLUD1 has recently been reported to exert non-catalytic regulatory functions through protein–protein interactions, including binding to the autophagy adaptor p62 (SQSTM1) to promote tumor cell survival under metabolic stress^16^. Thus, GLUD1 regulates the ARAF–MEK–ERK axis through both enzymatic and non-enzymatic mechanisms, with protein stabilization representing the dominant mode of action in detached ovarian cancer cells.

Within the canonical RAS–RAF–MEK–ERK signaling cascade, the RAF kinases ARAF, BRAF, and CRAF serve as central regulators of cell proliferation, differentiation, and survival^17–19^. The stability and integrity of RAF proteins are tightly controlled and are essential for signaling fidelity^20–23^. Although HSP90 is a well-established chaperone required for stabilization of RAF family kinases^20^, overexpression of HSP90 did not rescue the reduction in ARAF protein levels caused by GLUD1 depletion (Supplementary Fig. 7A). Instead, silencing GLUD1 selectively reduced ARAF protein abundance without affecting BRAF or CRAF, indicating that GLUD1 stabilizes ARAF at the post-translational level through an HSP90-independent mechanism. Consistently, GLUD1 depletion shortened the half-life of ARAF and increased its ubiquitination, supporting a model in which GLUD1 limits ubiquitin–proteasome–mediated degradation of ARAF. Moreover, restoration of ARAF expression in GLUD1-deficient cells partially rescued anoikis resistance under detachment conditions and restored migratory capacity, providing functional evidence that ARAF stabilization is a key downstream effector of GLUD1-mediated survival and metastatic behavior. In addition, GLUD1 depletion did not appreciably alter FAK, AMPK, or CREB signaling, suggesting that these pathways are unlikely to play dominant roles in GLUD1-regulated anoikis resistance (Supplementary Fig. 7B). Collectively, these data support a specific, post-translational, and HSP90-independent role for GLUD1 in maintaining ARAF stability and downstream ERK signaling.

Functionally, the GLUD1–ARAF–MEK/ERK axis confers a survival advantage to detached ovarian cancer cells by suppressing anoikis and facilitating metastatic progression. The prominence of this regulatory mechanism under suspension conditions highlights a context-specific requirement for ARAF-dependent ERK signaling during transcoelomic dissemination. The clinical relevance of this axis is further supported by the positive correlation between GLUD1 and ARAF expression observed in epithelial ovarian cancer specimens. However, due to the relatively limited patient sample size, it is necessary to validate these findings in larger, multicenter independent patient cohorts in future studies. Collectively, these findings identify GLUD1 as a previously unrecognized modulator of ARAF and establish a mechanistic link between glutamine metabolism and RAF–MEK–ERK signaling in epithelial ovarian cancer.

Beyond its mechanistic significance, identification of the GLUD1–ARAF–MEK/ERK axis also raises the possibility of therapeutic intervention in epithelial ovarian cancer. Pharmacologic inhibition of GLUD1, including agents such as R162, as well as clinically available MEK or ERK inhibitors, may disrupt the detachment-survival program that drives peritoneal metastasis^11^^,24^. However, GLUD1 is a central metabolic enzyme, and its dual metabolic and non-enzymatic functions introduce important complexity for therapeutic targeting. An unresolved question is how GLUD1 coordinates its enzymatic and non-enzymatic activities in different cellular contexts, and under what conditions each function predominates. Although our data support a role for GLUD1 in ARAF stabilization that cannot be fully explained by its metabolic output alone, the molecular mechanisms that govern the balance between GLUD1’s catalytic and non-canonical scaffolding functions remain to be defined. Further studies will be required to elucidate the regulatory cues, subcellular compartmentalization, or interaction networks that determine GLUD1 functional engagement in ovarian cancer.

## Methods

### In silico analysis

Relevant datasets were retrieved from the Gene Expression Omnibus (GEO) database (http://www.ncbi.nlm.nih.gov/geo/). Based on these criteria, three datasets (GSE26712, GSE27651, and GSE40595) were selected for further analysis. Differential expression analysis was conducted using the limma package in R (v3.46.0), applying thresholds of |log₂FC | > 0.7 and (FDR) < 0.05. In addition, volcano plots were generated to visualize gene distribution based on statistical significance and fold change, enabling intuitive comparison of expression patterns. A total of 422 anoikis-related genes and 709 amino acid metabolism-related genes were retrieved from published literature^25^. We performed intersection analysis using Venn diagrams to identify the overlapping genes, which are considered core genes involved in both anoikis and amino acid metabolism. Kaplan-Meier analysis (https://kmplot.com) was used to assess the prognostic significance in ovarian cancer.

### Clinical tissue specimens

Ovarian cancer tissues were collected from 60 epithelial ovarian cancer (EOC) patients at Guangdong Provincial People’s Hospital. Written informed consent was obtained from all participants, and the study was approved by the Ethics Committee of Guangdong Provincial People’s Hospital (Approval No. KY-Z-2020-518-03), in accordance with the Declaration of Helsinki. In addition, formalin-fixed, paraffin-embedded ovarian cancer tissue microarrays (TMAs) were obtained from the National Engineering Center for Biochip (Outdo Biotech, Shanghai, China). Use of these TMAs was approved by the Outdo Biotech Clinical Research Ethics Committee (Approval No. SHYJS-CP-1804029). Informed consent for TMA samples was obtained by the provider or waived due to the use of anonymized archival specimens, in accordance with institutional guidelines. The TMAs included 4 cases of adjacent non-tumor tissue, 134 cases of primary ovarian cancer, and 12 cases of metastatic tumors. Corresponding clinical information is provided in Supplementary Table 1.

### Cell culture

The human embryonic kidney cell line HEK293T and human ovarian cancer cell lines SKOV3, OVCAR3, HO8910PM, ES-2, A2780, and Caov-3 were obtained from the American Type Culture Collection (ATCC, Manassas, VA, USA). Cells were cultured in Dulbecco’s Modified Eagle Medium (DMEM; GIBCO) supplemented with 10% fetal bovine serum (FBS; ExCell) and 1% penicillin-streptomycin. All cells were maintained at 37 °C in a humidified incubator with 5% CO₂.

### Lentiviral transduction and plasmid transfection

Lentiviral vectors encoding full length GLUD1 or short hairpin RNAs targeting GLUD1 were co-transfected with packaging plasmids (psPAX2 and pMD2.G) into HEK293T cells to generate lentiviral particles. Viral supernatants were collected 48 hours after transfection, filtered, and concentrated as needed. OVCAR3 and SKOV3 cells were infected with the lentiviruses in the presence of polybrene (8 μg/mL), followed by puromycin selection (1 μg/mL) to establish stable GLUD1-overexpressing and GLUD1-silenced cell lines. The shRNA targeting sequence for GLUD1 was GCTACCTGGGCGAAGCGCTGT. In addition, plasmids encoding full-length ARAF and HSP90 were purchased from the MiaoLing Plasmid Platform and transfected into cells as indicated.

### Anoikis induction

Anoikis was induced by culturing cells in ultra-low attachment plates. Cells were harvested, washed, and seeded in serum containing medium under suspension conditions for the indicated time points. Cells cultured on standard tissue culture plates were used as attached controls.

### Cell viability and apoptosis assays

Cell viability under detached conditions was assessed using Calcein AM and propidium iodide staining according to the manufacturer’s instructions. Apoptosis was further quantified using Annexin V FITC and propidium iodide staining followed by flow cytometry analysis. For all assays, three independent biological replicates were performed.

### Immunofluorescence microscopy

Cells grown on glass coverslips were incubated with 100 nM MitoTracker Deep Red (Invitrogen) for 20 min at 37 °C. After staining, cells were fixed with paraformaldehyde, permeabilized, and blocked in PBS containing 5% BSA. Cells were then incubated with primary antibodies against GLUD1 (mouse, 1:200) and ARAF (rabbit, 1:200), followed by Alexa Fluor 488–conjugated anti-mouse IgG and Alexa Fluor 555–conjugated anti-rabbit IgG secondary antibodies (1:500), each for 1 hour at room temperature. Nuclei were counterstained with DAPI, and coverslips were mounted using antifade mounting medium. Images were acquired using a confocal fluorescence microscope.

### Western blot analysis

Western blotting was performed using primary antibodies from multiple sources. Antibodies against GLUD1 (1:1000, Cat# 14299-1-AP), GAPDH (1:5000, Cat# 60004-1-Ig), MEK (1:1000, Cat# 11049-1-AP), BRAF (1:1000, Cat# 20899-1-AP) antibody, CRAF (1:1000, Cat# 66592-1-Ig) antibody, HSP90(1:1000, Cat# 13171-1-AP) antibody, and Flag-tag (1:1000, Cat# 20543-1-AP) were obtained from Proteintech Group (Chicago, IL, USA). Antibodies against phospho-MEK (p-MEK) (1:1000, Cat# 9154 T), ERK (1:1000, Cat# 4695 T), phospho-ERK (p-ERK) (1:1000, Cat# 4370 T), BIM(1:1000, Cat# 2933 T) antibody, cl-caspase 3(1:1000, Cat# 9664S) antibody, FAK(1:1000, Cat# 3285 T) antibody, p-FAK(1:1000, Cat# 8556 T) antibody, AMPK(1:1000, Cat# 5831 T) antibody, p-AMPK(1:1000, Cat# 2535 T) antibody, CREB(1:1000, Cat# 9197 T) antibody, p-CREB(1:1000, Cat# 9198 T), and BCL-2 (1:1000, Cat# 15071 T) were purchased from Cell Signaling Technology (Danvers, MA, USA). The ARAF antibody (1:1000, Cat# ab200653) was obtained from Abcam (Cambridge, UK). antibody IRDye® 800CW Goat anti-Rabbit IgG (1:5000, Cat# 926-32211) and IRDye® 680RD Goat anti-Mouse IgG (1:5000, Cat# 926-68070) secondary antibodies were purchased from LI-COR Biosciences (Lincoln, NE, USA). Protein bands were visualized using the Odyssey CLx Imaging System and quantified with Image Studio Lite software (version 4.0; LI-COR Biosciences).

### Co-immunoprecipitation (Co-IP) assay

Co-IP assays were performed as previously described. Briefly, cells were lysed in IP lysis buffer supplemented with a protease inhibitor cocktail. The total lysates were incubated overnight at 4 °C with pre-conjugated magnetic beads (MedChemExpress, USA) on a rotating wheel. The following day, supernatants were carefully removed using a magnetic stand, and the protein-bound beads were washed six times with wash buffer. Proteins were eluted by adding loading buffer and heating at 100 °C for 10 min, followed by immunoblotting analysis.

### Ubiquitination assay

OVCAR3 and SKOV3 cells were transfected with plasmids expressing Myc-ARAF, Flag-ubiquitin (Flag-Ub), and GLUD1 as indicated. Cells were treated with MG132 (20 μM) for 6 h to inhibit proteasomal degradation and allow ubiquitinated proteins to accumulate. Cell lysates were prepared and subjected to immunoprecipitation using an anti-Myc antibody to pull down Myc-ARAF. The immunoprecipitants were then analyzed by western blotting with an anti-Flag antibody to detect ubiquitinated ARAF.

### Immunohistochemistry and TUNEL staining

Tissue sections were processed and stained according to standard protocols at the Biopathology Institute (Servicebio, Wuhan, Hubei, China) using primary antibodies against GLUD1 (1:1000), ARAF (1:100), and Ki-67 (1:200). Immunostaining intensity was scored as follows^26^: negative (0), weak (1), moderate (2), and strong (3). The extent of staining was evaluated based on the percentage of positively stained cells: 0 (<10%), 1 (10–25%), 2 (25–50%), 3 (50–75%), and 4 (>75%). In the xenograft mouse model, mice were sacrificed by cervical dislocation. Tumors were excised, fixed in 4% formaldehyde, embedded in paraffin, and subjected to hematoxylin and eosin (H&E) staining at the Biopathology Research Institute (Servicebio). Images of H&E-stained sections were captured using a Leica DM6B microscope (Leica, Wetzlar, Hessen, Germany). Apoptosis in tumor tissues was assessed using a TUNEL assay kit according to the manufacturer’s instructions, and apoptotic indices were calculated as the percentage of TUNEL positive cells.

### Cell migration assays

Cell migration was evaluated using Transwell chambers and wound healing assays. For Transwell assays, cells were seeded into the upper chamber in serum free medium, and medium containing 10 percent fetal bovine serum was added to the lower chamber as a chemoattractant. After incubation, migrated cells were fixed, stained, and quantified. Wound healing assays were performed by creating a linear scratch and monitoring wound closure over time. All migration assays were performed in three independent experiments.

### Intracellular metabolite measurements

Intracellular α-KG levels were quantified using a commercial α-ketoglutarate assay kit (Sigma-Aldrich) following the manufacturer’s instructions. Briefly, 2 × 10^6 cells were homogenized in PBS, and the supernatant was collected after centrifugation. Proteins were removed using 10-kDa Amicon Ultra centrifugal filters (Millipore), and the resulting flow-through containing small metabolites was used for α-KG measurement.

### Animal experiments

Xenograft tumor experiments were approved by the Institutional Animal Care and Use Committee of Guangdong Provincial People’s Hospital (Guangdong Academy of Medical Sciences, Southern Medical University; Approved No.KY-Z-2020-518-03). To ensure adequate statistical power, six female BALB/c-nu mice (6 weeks old) were used per group, and mice were randomly assigned to experimental groups^27–29^. For the intraperitoneal metastasis model, a single-cell suspension containing 2 × 10⁶ cells was injected into the peritoneal cavity of BALB/c-nu mice. In survival studies, mice that met predefined endpoint criteria such as sustained weight loss, respiratory distress, or irreversible recumbency were euthanized by cervical dislocation without anesthesia, in accordance with institutional animal care guidelines. Tumors were harvested and fixed in paraformaldehyde. Visible tumor nodules were counted and fixed tumor tissues were subsequently embedded in paraffin, sectioned, and stained with hematoxylin and eosin (H&E) for histological analysis.

### Statistical analysis

Functional analysis was performed using the SPSS software package (version 24.0, IBM SPSS, IL, USA) and GraphPad Prism (version 8.0, GraphPad Software, CA, USA). We used Student’s *t* test to examine the difference in means between two groups, and One-way ANOVA to examine the difference between multiple groups. A Chi-square test was used for correlation analysis between gene expression and clinicopathological features. The Kaplan–Meier method was used to test the overall survival between different groups. A *p* value < 0.05 was considered a statistically significant difference.

## Supplementary information

Supplementary Material

Supplementary information

## Data Availability

All data supporting the findings of this study are presented in the article and the Supplementary Materials. Our study does not generate any new datasets. The datasets presented in this study can be found from the following links: TCGA (https://portal.gdc.cancer.gov/), GSE26712, GSE27651 GSE40595. All other raw data are available upon reasonable request from the corresponding author.

## Code availability

The methods described in this manuscript have been used the public available limma package in R (v3.46.0) for differential gene expression analysis. No custom algorithm generated in this manuscript.
