# Supplementary material for: GLUD1 supports ovarian cancer progression by counteracting anoikis via ARAF/MEK/ERK signaling
Source: NPJ Precis Oncol. 2026 Mar 5;10:151. doi: 10.1038/s41698-026-01349-6 (PMC13079734; doi:10.1038/s41698-026-01349-6)

# Supplementary Material

## GLUD1 Supports Ovarian Cancer Progression by Counteracting Anoikis via ARAF/MEK/ERK Signaling

Huolun Feng[#1,2], Yanzhen Chen[#3], Geyan Wu[#4], Zhentao Zhang[5], Hongkun Lai[2], Changnian Yang[5], Shaofen Zhang[3], Yongqing Lin[3], Yingqi Liu[5], Haiyan Ye[*3], Shanshan Wu[*6], Lixue Cao[*7]

## Supplementary Figures

### Supplementary Figure 1

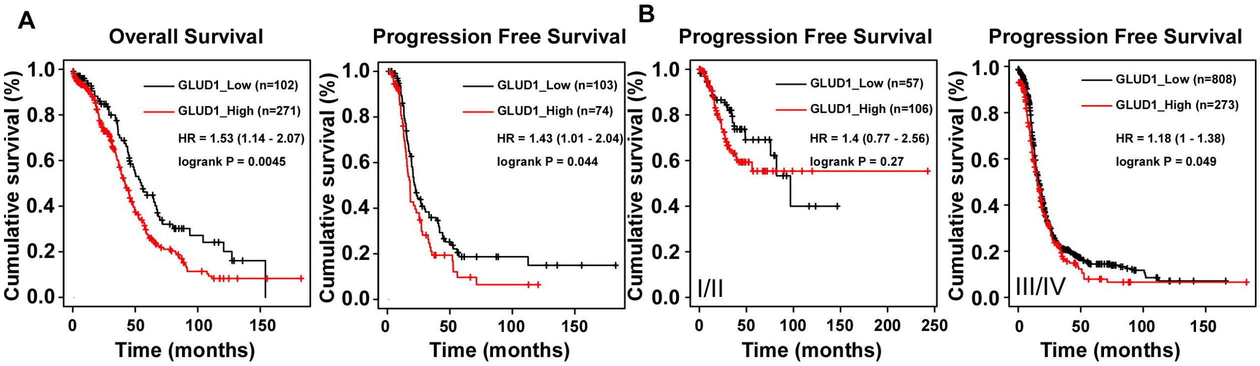


**Supplementary Figure 1: Elevated GLUD1 expression is associated with poor prognosis in EOC patients.** (A) Kaplan–Meier progression free survival curves of epithelial ovarian cancer patients stratified by GLUD1 expression in early-stage disease (FIGO I–II).

(B) Kaplan–Meier progression free survival curves of epithelial ovarian cancer patients stratified by GLUD1 expression in advanced stage disease (FIGO III–IV).

### Supplementary Figure 2

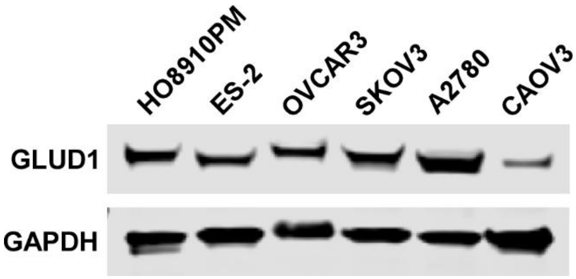


**Supplementary Figure 2: GLUD1 expression across ovarian cancer cell lines** Western blot analysis of GLUD1 protein expression in the indicated ovarian cancer cell lines. GAPDH was used as a loading control.

**Supplementary Figure 3**

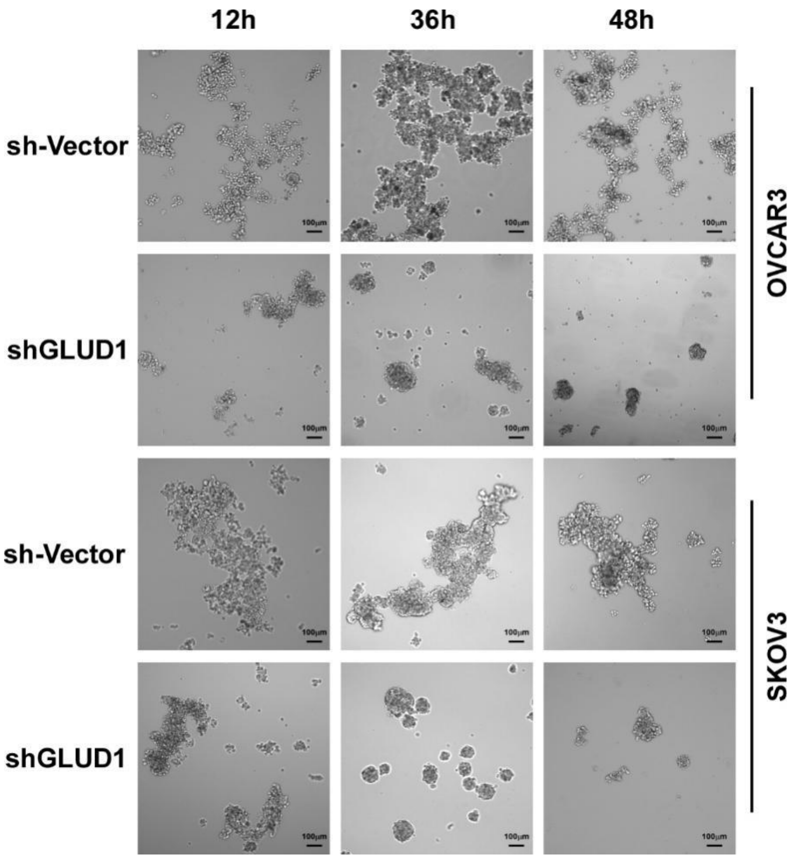


**Supplementary Figure 3: GLUD1 knockdown suppresses spheroid growth under detached conditions.** Representative images of tumor spheroids formed by control and GLUD1 silenced ovarian cancer cells cultured under low attachment conditions at the indicated time points. Scale bar: 100 µm.

**Supplementary Figure 4**

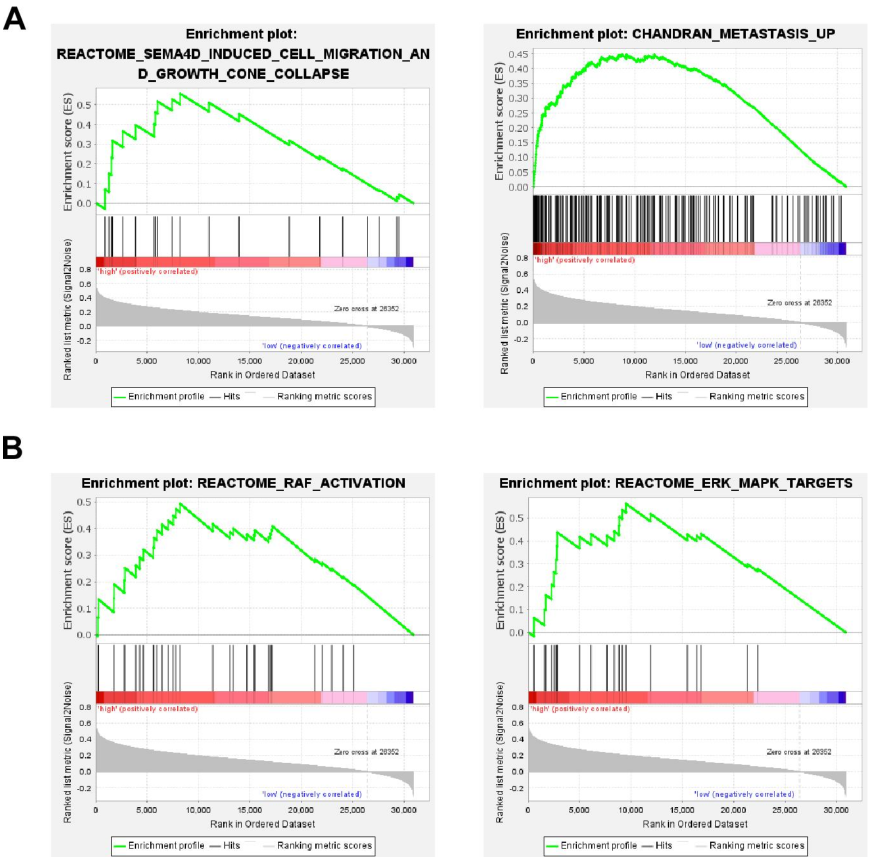


**Supplementary Figure 4: Association of GLUD1 expression with metastatic and signaling gene signatures. (A)** Gene set enrichment analysis showing enrichment of migration and metastasis related gene signatures in samples with high GLUD1 expression. (**B**) Gene set enrichment analysis showing enrichment of RAF/MEK/ERK signaling pathways in samples with high GLUD1 expression. based on TCGA data.

**Supplementary Figure 5**

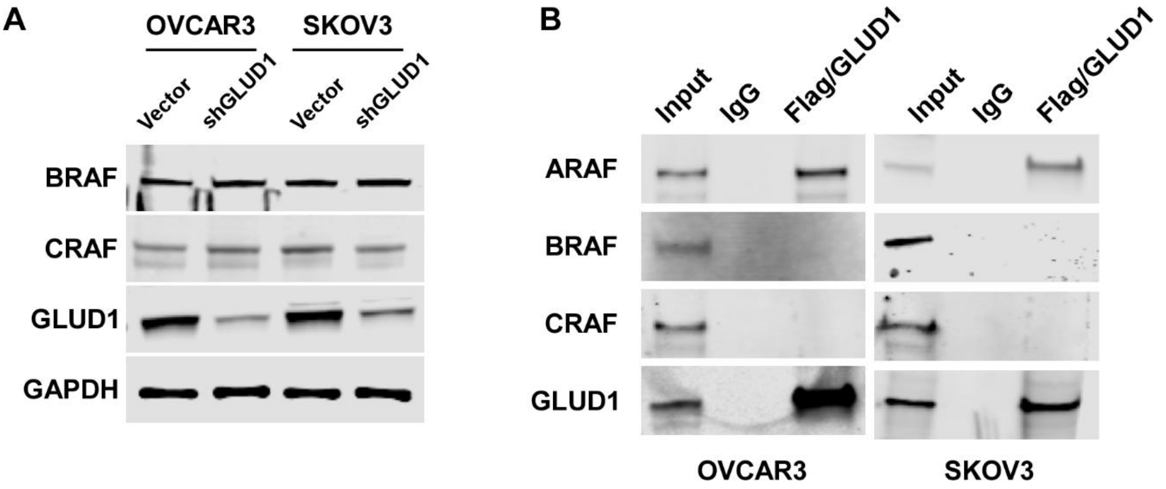


**Supplementary Figure 5: GLUD1 selectively interacts with ARAF but not BRAF or CRAF in EOC cells.** (A) Western blot analysis of BRAF and CRAF protein levels following GLUD1 knockdown. (B) Co immunoprecipitation analysis assessing the interaction between GLUD1 and RAF isoforms ARAF, BRAF, and CRAF in ovarian cancer cells.

**Supplementary Figure 6**

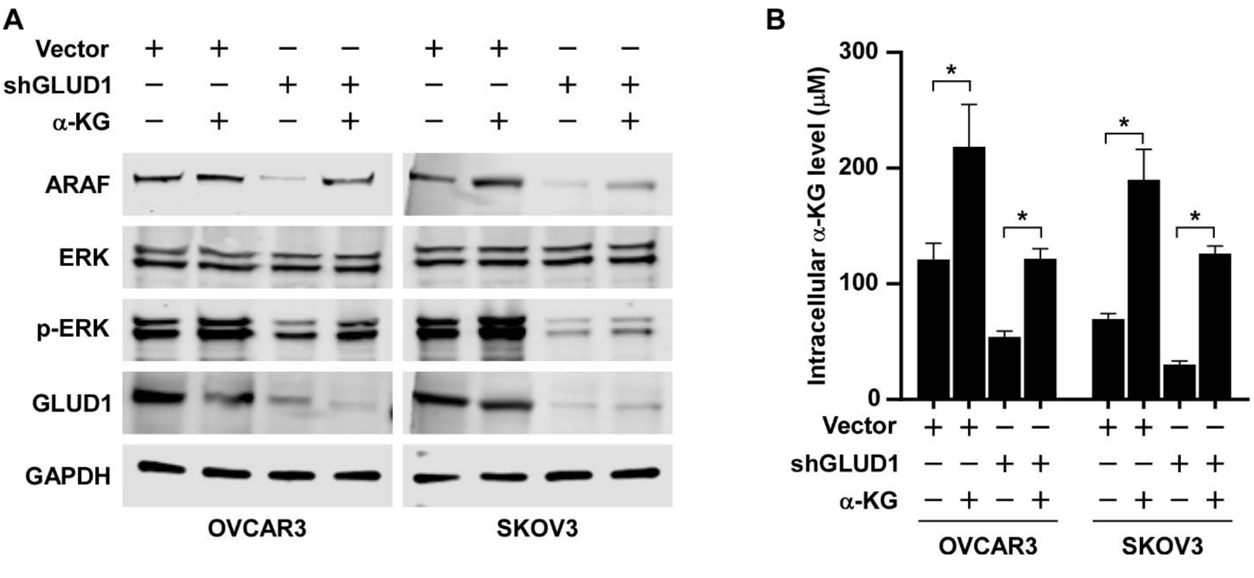


**Supplementary Figure 6: Contribution of GLUD1 metabolic activity to ARAF stability and ERK activation.** (A) Western blot analysis showing the effects of α-KG supplementation on ARAF stability and MEK/ERK signaling in OVCAR3 and SKOV3 cells. (B) Quantification of

intracellular α-KG concentrations in OVCAR3 and SKOV3 cells under the indicated conditions. *$p < 0.05$.

**Supplementary Figure 7**

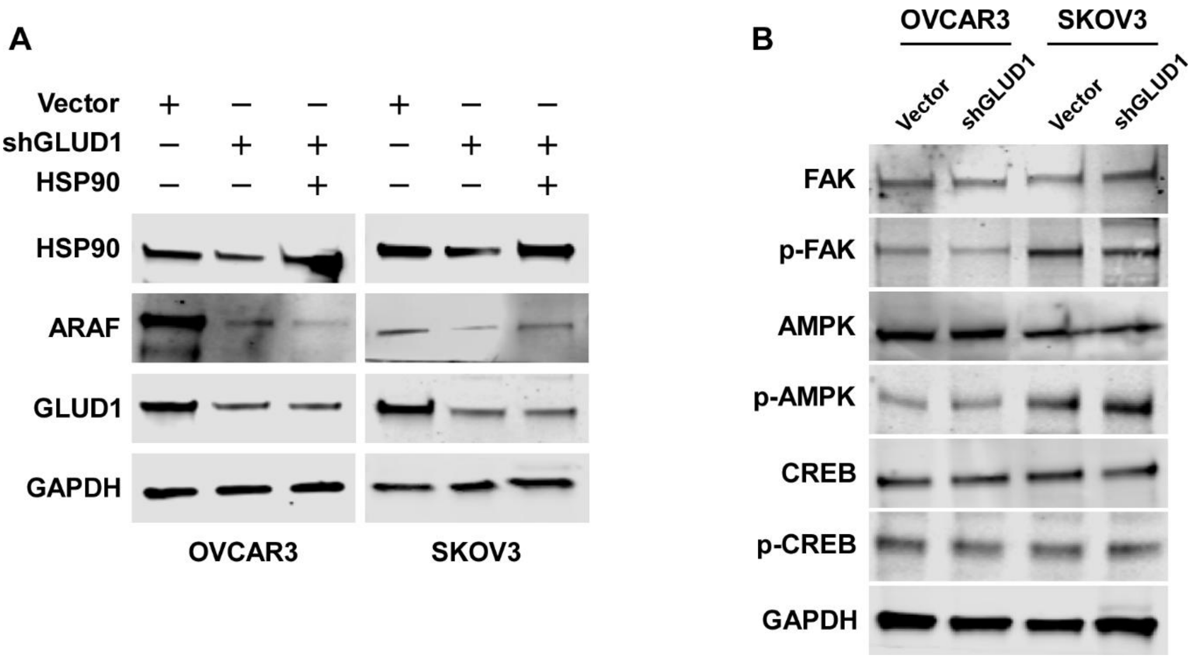


**Supplementary Figure 7** GLUD1 regulates ARAF stability independently of HSP90 and does not affect FAK, AMPK and CREB signaling. (A) Western blot analysis showing the effects of GLUD1 knockdown and HSP90 overexpression on ARAF protein levels in OVCAR3 and SKOV3 cells. (B) Western blot analysis of FAK, AMPK, and CREB signaling pathways in OVCAR3 and SKOV3 cells upon GLUD1 knockdown.

**Supplementary Table 1**. Clinicopathological characteristics and GLUD1 expression in 146 patients with ovarian cancer.

| Clinical features | Number | GLUD1 expression | | *p* values |
|---|---|---|---|---|
| | | Low(n=63) | High (n=83) | |
| **Age (years)** | 146 | | | |
| ≤ 62 | 122 | 52 | 70 | 0.772 |
| > 62 | 24 | 11 | 13 | |
| **Histological type** | 146 | | | |
| Serous | 80 | 36 | 44 | |
| Endometrioid | 17 | 8 | 9 | |
| Clear cell | 3 | 1 | 2 | 0.885 |
| Mucinous | 33 | 14 | 19 | |
| Undifferentiated | 13 | 4 | 9 | |
| **FIGO stage** | 146 | | | |
| I/II | 42 | 20 | 22 | 0.488 |
| III/IV | 104 | 43 | 61 | |
| **Histologic grade** | 121 | | | |
| 1 | 14 | 6 | 8 | |
| 2 | 18 | 9 | 9 | 0.698 |
| 3 | 89 | 35 | 54 | |
| **Recurrence** | 146 | | | |
| Yes | 119 | 49 | 70 | 0.312 |
| No | 27 | 14 | 13 | |
| **Vital status** | 146 | | | |
| Alive | 69 | 38 | 31 | 0.006 |
| Dead | 77 | 25 | 52 | |

**Supplementary Table 2**. Univariate and multivariate analysis of different prognostic parameters in patients with ovarian cancer by Cox-regression analysis.

| | Univariate analysis | | Multivariate analysis | |
|---|---|---|---|---|
| | ***p*** | **Hazard ratio (95% CI)** | **P** | **Hazard ratio (95% CI)** |
| **Age (years)** | 0.013 | 1.026 (1.005-1.047) | 0.156 | 1.013 (0.995-1.032) |
| **Histological type** | | | | |
| Serous | 0.776 | 1.122 (0.508-2.481) | | |
| Endometrioid | 0.235 | 0.516 (0.173-1.537) | | |
| Clear cell | 0.75 | 1.292 (0.268-6.229) | | |
| Mucinous | 0.178 | 0.527 (0.207-1.339) | | |
| Undifferentiated | Ref. | | | |
| **FIGO stage** | < 0.001 | 11.115 (4.056-30.460) | < 0.001 | 9.903 (3.593-27.289) |
| **GLUD1 expression** | 0.008 | 1.12 (1.03-1.217) | 0.028 | 1.098 (1.013-1.191) |

## Uncropped images of the WB

### Figure 1E

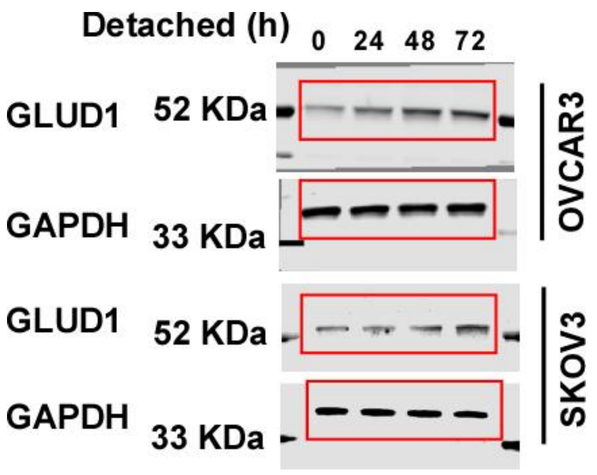


### Figure 2A

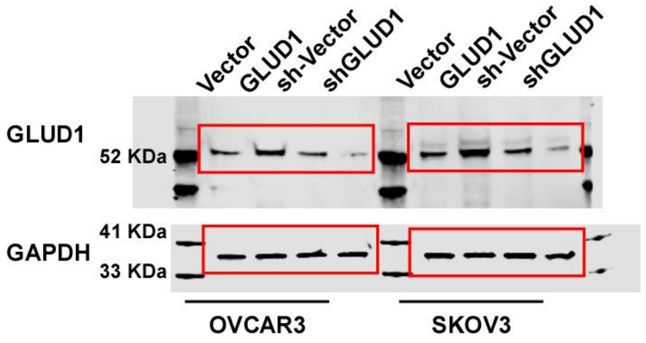

# Figure 4C-G

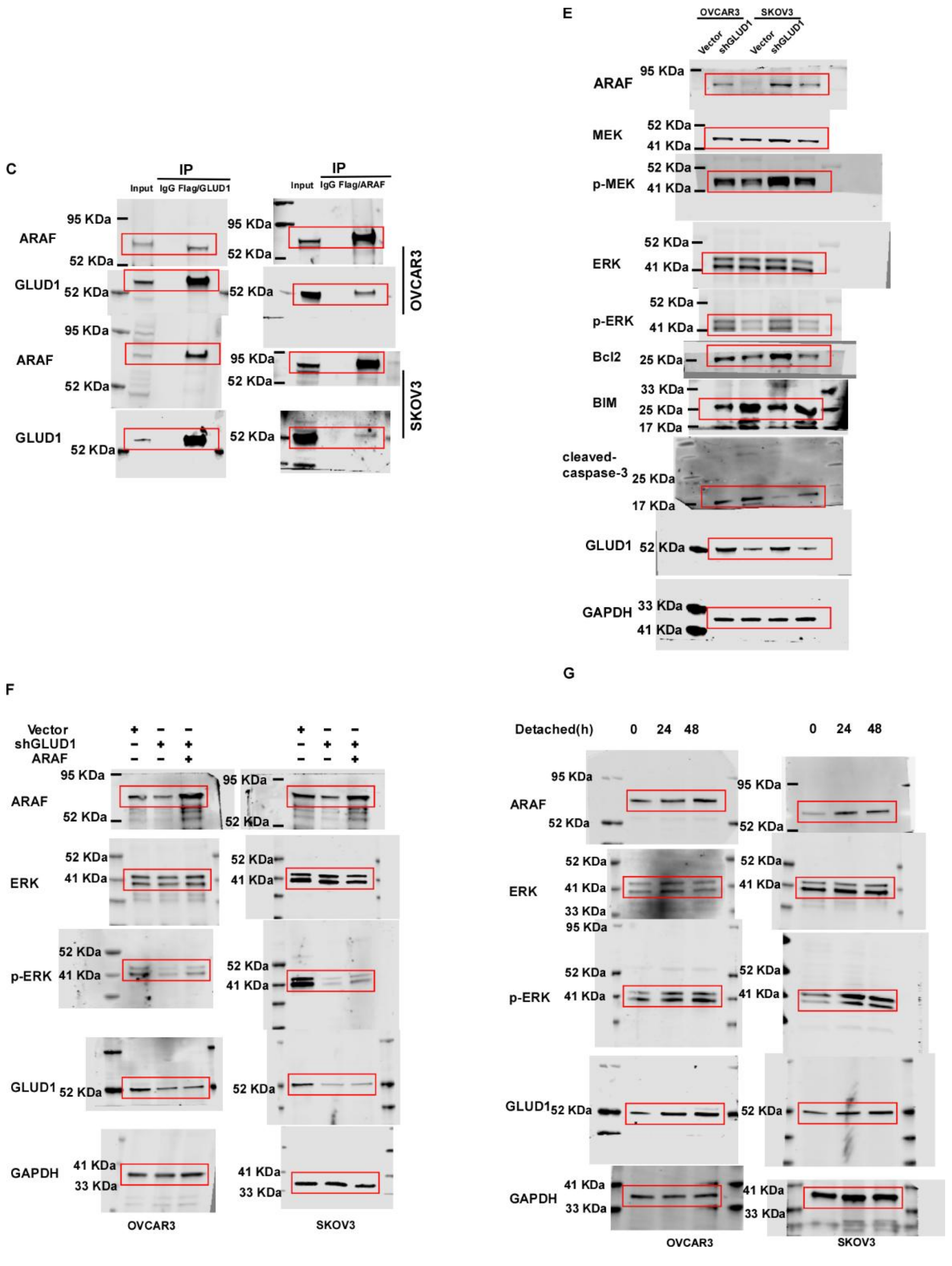

C
IP
Input IgG Flag/GLUD1
Input IgG Flag/ARAF
ARAF
GLUD1
OVCAR3
SKOV3
E
OVCAR3 SKOV3
Vector shGLUD1 Vector shGLUD1
ARAF
MEK
p-MEK
ERK
p-ERK
Bcl2
BIM
cleaved-caspase-3
GLUD1
GAPDH
F
Vector
shGLUD1
ARAF
G
Detached(h) 0 24 48
OVCAR3
SKOV3

## Figure 5A-C

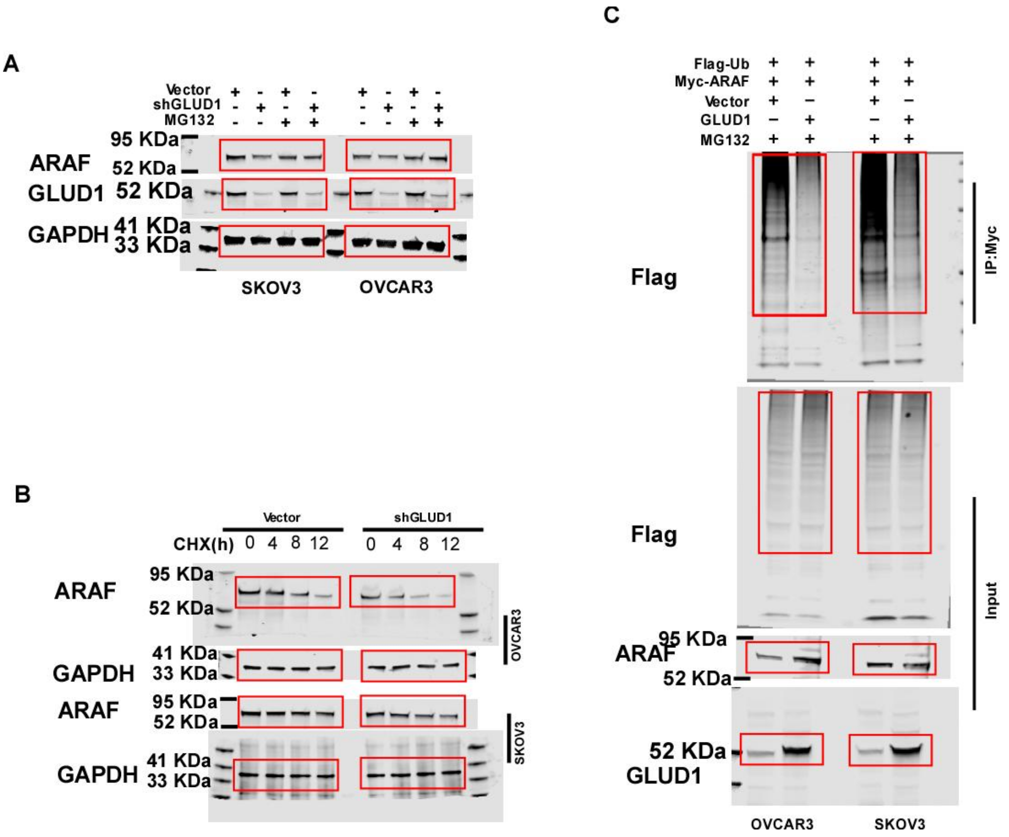


## Figure 6B

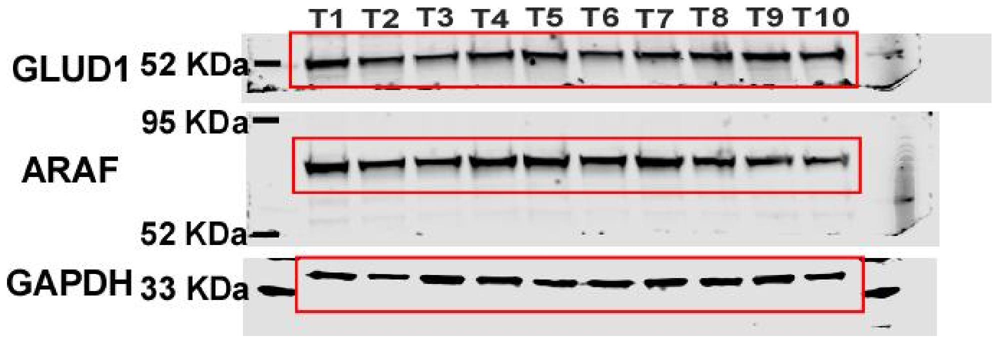


## Supplementary Figure 2

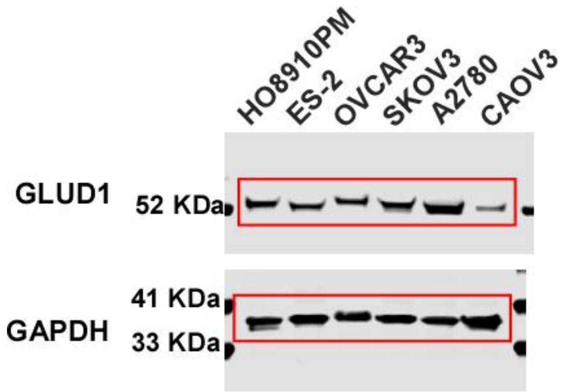

## Supplementary Figure 5A-B

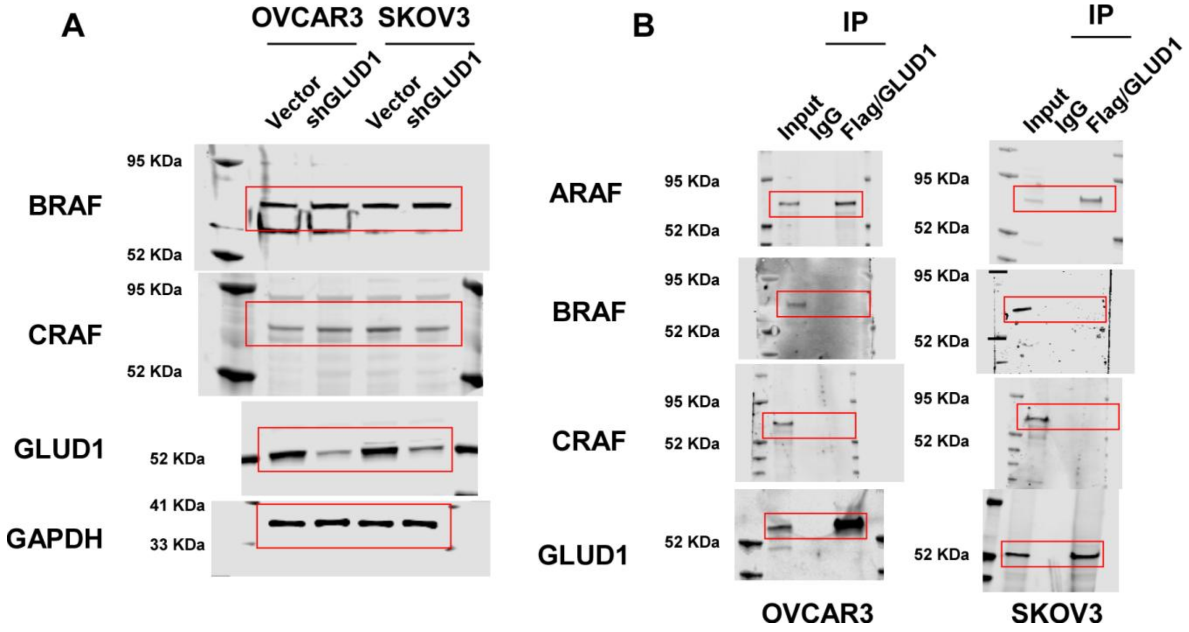


## Supplementary Figure 6A

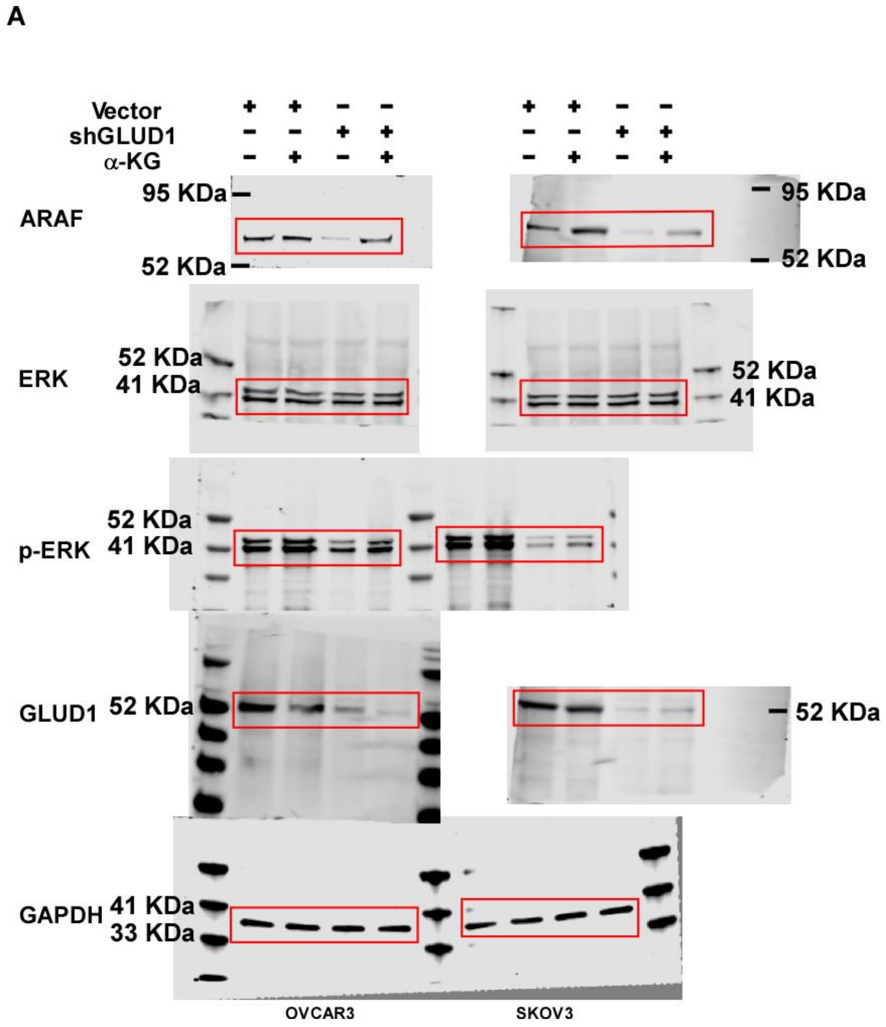

## Supplementary Figure 7A-B

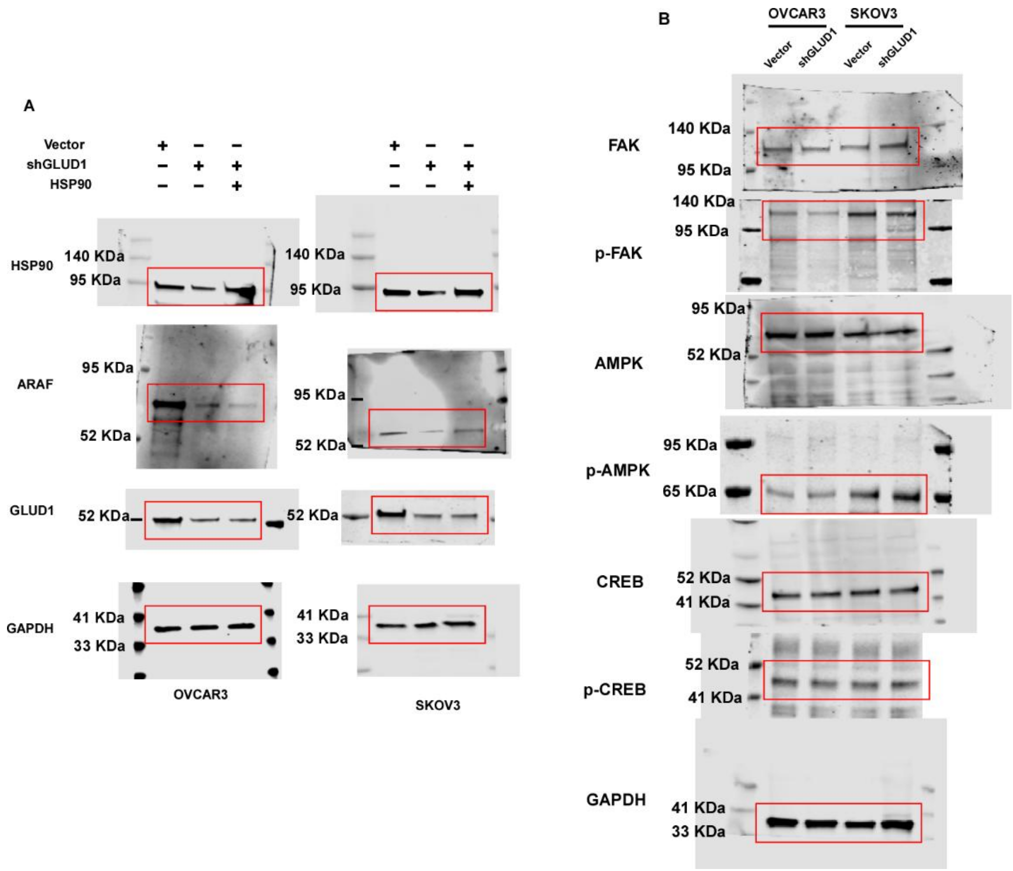

Supplement: Supplementary file 1 — Supplementary Material [file 41698_2026_1349_MOESM1_ESM.pdf]
